# Factors associated with knowledge and attitude towards maternity waiting homes among pregnant women: baseline results from a cluster-randomized trial in rural Ethiopia

**DOI:** 10.1038/s41598-023-39029-1

**Published:** 2023-07-22

**Authors:** Teklemariam Ergat Yarinbab, Hailay Abrha Gesesew, Margo Shawn Harrison, Tefera Belachew

**Affiliations:** 1 Department of Population and Family Health, Institute of Health, Jimma University, Jimma, Ethiopia; 2 Department of Epidemiology and Biostatistics, College of Health Sciences, Mizan Tepi University, Mizan Teferi, Ethiopia; 3 Research Center for Public Health, Equity and Human Flourishing, Torrens University Australia, Adelaide, Australia; 4 School of Public Health, College of Health Sciences, Mekelle University, Mekelle, Ethiopia; 5 Department of Gynecology and Obstetrics, School of Medicine, University of Colorado, Denver, CO USA; 6 Department of Nutrition and Dietetics, Institute of Health, Jimma University, Jimma, Ethiopia

**Keywords:** Health care, Medical research

## Abstract

Ethiopia has implemented maternity waiting homes over the last several decades; however, its utilization is low. This study aimed to assess the factors associated with knowledge of and attitude towards maternity waiting homes among pregnant women in rural Ethiopia. The baseline survey was conducted from September 15 to October 30, 2022, in rural Southern Ethiopia. Survey data were collected from 320 women in their second trimester of pregnancy. The data analysis was performed using SPSS version 25. The mean age of the participants was 27.79 (SD ± 6.242) years. Nearly two-thirds (57.5%) of the participants had no formal education and more than three-fourths (72.5%) were housewives. Only approximately one-fourth (23.75%) of the participants used maternity waiting homes. Furthermore, 33.75% had good knowledge, 28.75% had favorable attitudes, and around one-fourth (26.25%) had good male partner involvement. Age group 30 to 39 years (AOR 4.78, 95% CI 1.12–20.36), household income (AOR 6.41, 95% CI 2.78–14.81), having pregnancy intention (AOR 2.63, 95% CI 1.21–5.73), and history of obstetric complications (AOR 6.72, 95% CI 2.81–16.07) were significantly associated with good knowledge about maternity waiting homes. Similarly, age group 30 to 39 years (AOR 4.23, 95% CI 1.14–15.65), household income (AOR 7.12, 95% CI 3.26–15.55), having pregnancy intention (AOR 2.57, 95% CI 1.21–5.47), and history of obstetric complications (AOR 5.59, 95% CI 2.30–13.59) were significantly associated with favorable attitudes towards maternity waiting homes. Providing health education and promoting male partner participation through educating couples may improve women’s access to maternity waiting homes.

## Introduction

Globally, the maternal mortality ratio was estimated to be 223 per 100,000 live births in 2020 and majority (95%) of maternal deaths have been reported to occur in resource-limited settings^1^. Sub-Saharan Africa alone accounts for approximately three-fourths of maternal deaths, and the maternal mortality ratio in Ethiopia is estimated to be 401 per 100,000 live births^1,2^. The leading causes of maternal deaths are obstetric hemorrhage, hypertensive disorders in pregnancy, non-obstetric complications, pregnancy-related infections, and unsafe abortion^3,4^. Most maternal deaths owing to these causes are preventable by enabling timely access to obstetric care^2,5^. However, poor access to obstetric facilities due to long distances, lack of transportation, and unfavorable road conditions aggravated women’s inability to receive timely obstetric care^6,7^.

Increasing health facility deliveries is vital for reducing maternal and neonatal mortality^8^. Although health facility delivery has been promoted in Ethiopia, home delivery is still common, primarily in geographically isolated areas^9^. The 2019 Mini Ethiopian Demographic and Health Survey showed that 48% of live births were delivered in a health facility, and access to health facilities is mentioned to be more difficult in rural areas than in urban areas because of distance, scarce transport, and a lack of appropriate facilities^10^. Maternity waiting home (MWH) has been recognized as a strategy to improve maternal health outcomes by bringing women in hard-to-reach areas closer to health facilities^11^. MWH is a shelter located near or in a health facility where women near their delivery date can stay and be transferred to obstetric facility shortly before childbirth or earlier if complications arise^12^. Ethiopia has implemented MWHs for the last several decades; however, its uptake is low^13,14^.

Lack of awareness about MWHs, women’s perceptions of the quality of care at MWHs, poor provider interactions with women staying at MWHs, poor physical aspects of MWHs, staff shortages, and household chores are some of the important barriers to staying at MWHs^15–17^. Use of MWHs also depends largely on male partners’ decisions^18,19^. Furthermore, a study from Northwest Ethiopia showed that antenatal care (ANC) visits, short distance to health facilities, women’s involvement in decision making, and MWH use were found to be associated with maternal knowledge, whereas higher education status, ANC visits, and short distance to the facility were associated with maternal attitudes towards MWHs^20^. However, further studies are required to have a comprehensive understanding about factors associated with women’s knowledge and attitudes regarding MWHs in rural Ethiopia.

## Methods and materials

### Study setting

The baseline data used in this analysis were collected from Ana Lemo and Gibe districts of Hadiya Zone of southern Ethiopia from September 15 to October 30, 2022. The two districts were purposefully selected based on the availability of functional MWHs. Based on the information we obtained from the Zonal health department, the total estimated population of the two districts was 265,000 in 2021. The two districts were divided into 52 clusters/kebeles (the smallest administrative units). One cluster has an average of 5000 population. There was one primary hospital, 10 health centers, 10 MWHs and 42 health posts in the two districts including two health centers and MWHs added from an adjacent district (Misha district). The livelihood of the population mainly depends on agriculture.

### Study participants

The study participants were pregnant women in the beginning of second trimesters of pregnancy (14–16 weeks of gestation) who were permanent residents of the study area, gave birth within the last 5 years preceding the current pregnancy, were living with their male partners at the time of data collection, were living ≥ 2 h of walking distance from the nearest health facility^21^ and had limited access to public transportation.

### Background about the trial

The data source for this analysis was a baseline survey conducted prior to intervention roll-out in an ongoing cluster-randomized trial aimed to evaluate the effectiveness of health education provided to couples on improving knowledge, attitude, and uptake of MWHs: group health education, home visits and provision of take-home print materials (ClinicalTrials.gov Identifier: NCT05015023). The intervention was provided at three contact points. The first contact point was the group health education at baseline whereas the second, and third contacts were home visits. Leaflets /print health messages were provided at each contact.

### Sample size calculations

The Hooper and Bourke method for cluster randomization studies of parallel arms with repeated cross-sections was used to calculate the sample size^22^. To illustrate within intra-cluster correlation coefficient (ICC) and between ICC, the technique comprises the measurement of two design effects, with the product of the two being used to inflate the sample size for individual randomization. The within ICC was the correlation between any two pregnant women in the same cluster, while the between ICC was the correlation between any two pregnant women in different clusters. The first design effect (dc) attributable to cluster randomization was measured using a within ICC of 0.05 obtained from a community-based cluster randomized trial in Ethiopia^23^. The design effect (d_c_) was calculated as:

$$\mathrm{dc}=1+\left(m-1\right)\rho ,$$

where m is the cluster size assumed to be 20 (i.e., the total number of pregnant women who were questioned in each cluster) and ρ was the within ICC.

The second design effect (d_r_) attributable to repeated evaluations (baseline/endline) was calculated by using the within ICC and a cluster autocorrelation coefficient (π) of 0.80^22^.

The second design effect (d_r_) was calculated as:

$${d}_{r}=\left(1-{r}^{2}\right),$$

where $$r=\left(\frac{m\rho \pi }{{d}_{c}}\right).$$

The required sample size was then calculated by multiplying the ‘sample size assuming individual randomization’ by both design effects (dc and dr). It was calculated as:

$$n = \left\lfloor {\frac{{\left( {a + b} \right)^{2} \times \left( {p_{1} q_{1} + p_{2} q_{2} } \right)}}{{\left( {|p_{1} - p_{2} |} \right)^{2} }}} \right\rfloor \times d_{c} d_{r} ,$$

where $n$ represents the sample size in each of the arms i.e., intervention and control. $a$ represents conventional multiplier (1.96) for alpha $\left(\alpha =0.05\right)$ and $b$ represents conventional multiplier (0.842) for power $(1-\beta =0.80)$. ${p}_{1}$ represents proportion of post-intervention users of MWH and ${q}_{1}$ represents proportion of post-intervention non-users of MWH. ${p}_{2}$ represents proportion (50%) of users of MWH taken from a study in Gurage Zone, Southwest Ethiopia^24^ and ${q}_{2}$ represents pre-intervention proportion of non-users of MWH. $|{p}_{1}-{p}_{2}|$ *an effect size*—was an absolute change in proportion of MWH utilization after intervention. It was estimated to be 20%.

In addition, the following parameters were considered: 95% CI 80% power, 1:1 allocation ratio of intervention to control, 10% potential loss to follow up, and tabulated sample size $({n}_{0}=199)$ required to detect a difference in two proportions at 5% significance level with 80% power in literature^25^. According to Hooper and Bourke, the number of clusters (K) for the sample was determined using the formula $K=\left({n}_{0}{d}_{c}{d}_{r}\right)/m$. The final sample size was calculated by substituting the specified values into the above formula. Hence, a total of 16 clusters were needed, with an approximated final sample size of 320. The two arms each had 160 eligible pregnant women (with their male partners).

### Ethics approval

Ethical approval letter was received from the IRB of Jimma University with reference number JUIRB-33/22, dated 09/02/2022. Consequently, a letter of permission was obtained from the Health Department of the Hadiya Zone, southern Ethiopia. The study participants were informed about the objective of the study, and written informed consent was obtained from each participant prior to the start of data collection. This study was registered in ClinicalTrials.gov with Identifier: NCT05015023. Link: https://clinicaltrials.gov/ct2/show/NCT05015023. All methods were performed in accordance with the relevant guidelines and regulations including the ethical guidelines of the Jimma University Ethical Review Committee and the declaration of Helsinki.

### Baseline survey

The baseline survey targeted 320 pregnant women from 16 clusters. There were 160 participants in each of the two arms with an average number of 20 participants in a cluster. Structured questionnaires were used to collect data through face-to-face interviews. The questionnaires were adapted from literature such as Ethiopian Demographic and Health Surveys and a global framework for assessing male involvement in maternal health^26,27^. The questionnaires were prepared in English, translated into local language (Amharic) and pre-tested. The pre-test was performed on 10% of the calculated sample size in Misha Woreda, a district outside of our study sites. Next, Cronbach’s Alpha was calculated using SPSS version 25 to test internal consistency of the knowledge, attitude and male partner involvement questions and Cronbach’s Alpha value greater than 0.7 was considered reliable. The data were collected on study variables such as socio-demographic characteristics, knowledge, attitude, and health service utilizations including MWH use (Additional file 1). The principal investigator and the other two field supervisors supervised the data collection. All the participants responded to the questionnaires.

### Variables of interest and measurement

The primary outcome variable was the self-reported proportion of MWH use for any pregnancy in the last 5 years whereas the secondary outcomes were knowledge of and attitudes toward MWHs. In this analysis, we aimed to assess the factors associated with knowledge and attitude towards MWHs. The proportion of MWH uptake was measured as the number of women who reported staying at MWH for at least one day for any pregnancy in the last 5 years divided by the number of women who gave birth during the same period (among the women enrolled in the trial) × 100. Knowledge was measured using 7 “Yes” or “No” questions. “Yes” was denoted by “1” and “No” was represented by “0”. Then the sum average for a respondent above the median (Y ≥ 4) was considered good knowledge and below 4 was considered poor knowledge. Attitude was measured using 5 points Likert scales (very disagree, disagree, neutral, agree, very agree). Five questions were used to measure attitude. Average scores above median were considered favorable attitude whereas average scores below median were considered unfavorable attitude.

To assess male partner involvement, we adopted a tool from a global framework for assessing male involvement in maternal health^27^. The global framework has 5 domains (involvement in communication, involvement in decision-making, practical support, physical support, and emotional support). Each of the 5 domains has 3–5 questions, with a total of 21 questions. We carefully adopted the tool to our context and collected the data. In this analysis, we selected 9 indicators (“Yes” or “No” questions) to measure the level of male partner involvement in maternal health. “Yes” scores above median (Y ≥ 5) were considered good male partner involvement and “Yes” scores below median (Y ≤ 4) were considered poor male involvement.

### Data analysis

The data analysis was conducted using SPSS Version 25. Descriptive statistics were performed and the results were presented using text, tables, and graphs. Bivariate and multivariate logistic regression analyses were conducted to identify the factors associated with pregnant women’s knowledge of and attitude toward MWHs. In logistic regressions, crude odds ratio (COR) and adjusted odds ratio (AOR) with the corresponding 95% confidence intervals were computed to show the strength of the association. Variables with p-value < 0.25 at bivariate analysis were fitted into the multivariate analysis. In the multivariate analysis, statistical significance was declared at a p-value of < 0.05. The Hosmer–Lemeshow test was used to check the goodness of fit for the logistic regression.

## Result

### Sociodemographic characteristics

Three hundred-twenty participants were included in the study. Nearly three-fourths (68.75%) of the participants were aged 20–29 years, and the mean age was 27.79 (SD 6.242) years. About half (53.75%) were from the ethnic group of Hadiya. One hundred eighty-four (57.5%) participants had no formal education, and more than three-fourths (72.5%) were housewives (Table 1).

**Table 1:** Sociodemographic characteristics of the study participants in rural southern Ethiopia, 2022.

Variables	Category	Frequency (n)	Percent (%)
Age (years)	20–29	220	68.75
	30–39	76	23.75
	≥ 40	24	7.5
Ethnic group	Hadiya	172	53.75
	Silte	52	16.25
	Gurage	72	22.5
	Others	24	7.5
Religion	Protestant	172	53.75
	Orthodox	68	21.25
	Muslim	56	17.5
	Others	24	7.5
Educational status	No formal education	184	57.5
	Grades 1–8	136	42.5
Occupation	Housewife	232	72.5
	Merchant	76	23.25
	Student	12	3.75
Household income	Less than 7985 ETB/month^a^	272	85
	≥ 7985 ETB/month	48	15

^a^Household income cut-off point: Anker National Living Household Income Reference Value for 2021 for rural Ethiopia is Birr 7985 per month.

### Obstetric related factors

Two hundred thirty-two (72.5%) and 224 (70%) participants reported parity and gravidity of four or fewer, respectively. Two hundred and seventy-two (85%) participants reported planned pregnancies, 40 (12.5%) reported gestational age at birth less than or equal to 37 weeks, and 88 (27.5%) reported a history of obstetric complications. Most of the deliveries (83.75%) were normal vaginal deliveries, whereas instrumental assisted and cesarean section births were 12.5% and 3.75%, respectively. Stillbirth was reported by 36 (11.25%) respondents (Table 2).

**Table 2:** Obstetrics related factors of the study participants in rural southern Ethiopia, 2022.

Variables	Category	Frequency (n)	Percent (%)
Parity	≤ 2	132	41.25
	3–4	100	31.25
	≥ 5	88	27.5
Gravidity	≤ 2	140	43.75
	3–4	84	26.25
	≥ 5	96	30
Pregnancy intention	Wanted	272	85
	Unwanted	48	15
Gestational age at birth	> 37 weeks	280	87.5
	≤ 37 weeks	40	12.5
Mode of delivery	Spontaneous vaginal delivery	268	83.75
	Instrumental delivery	40	12.5
	Cesarean section delivery	12	3.75
History of complications	Yes	88	27.5
	No	232	72.5
Birth outcome	Live birth	284	88.75
	Stillbirth	36	11.25

### Maternal health service-related factors

The proportion of participants who received ANC (at least once) was 124 (38.75%), and 76 (23.75%) used MWH. There were 84 (26.25%) institutional deliveries and 68 (21.25%) PNC visits within two weeks after delivery. Eighty-four (26.25%) participants were assessed to have good involvement of male partners in maternal health (Table 3).

**Table 3:** Health service-related factors of participants in rural southern Ethiopia, 2022.

Variables	Category	Frequency (n)	Percent (%)
ANC visit (at least once)	Yes	124	38.75
	No	196	61.25
Used MWH	Yes	76	23.75
	No	244	76.25
Delivered at health facility	Yes	84	26.25
	No	236	73.75
Visited health facility for PNC (within 2 weeks after delivery)	Yes	68	21.25
	No	252	78.75
Male-partner involvement	Good	84	26.25
	Poor	236	73.75

### Knowledge and attitude related to MWHs

About 108 (33.75%) of the participants demonstrated good knowledge of MWHs and 92 (28.75%) had favorable attitude towards MWHs.

### Factors associated with knowledge of and attitude towards MWHs

In the bivariate logistic regression, household income, pregnancy intention, and history of obstetric complications were associated with both knowledge and attitude towards MWHs. Educational status was inversely associated with knowledge and attitude whereas age was not associated with either knowledge or attitude towards MWHs. In the multivariate regression, age (30–39 years), household income, pregnancy intention (having wanted pregnancy), and history of obstetric complications were statistically associated with good knowledge of and favorable attitude towards MWHs.

Consequently, women in the age group 30–36 years (AOR 4.78, 95% CI 1.12–20.36) were nearly 5 times more likely to have good knowledge about MWHs compared to women in the age group ≥ 40 years. Women with average household monthly income below 7985 ETB (AOR 6.41, 95% CI 2.78–14.81) were 6 times more likely to have good knowledge of MWHs compared to women with household income above 7985 ETB per month. Likewise, women having wanted pregnancy (AOR 2.63, 95% CI 1.21–5.73) and those having a history of obstetric complications (AOR 6.72, 95% CI 2.81–16.07) were nearly 3 times and 7 times more likely to have good knowledge of MWHs compared to women having unwanted pregnancy and those had no history of obstetric complications, respectively (Table 4).

**Table 4:** Factors associated with women’s knowledge of MWHs in rural southern Ethiopia, 2022.

Variables	Level of Knowledge		Crude OR (95% CI)	Adjusted OR (95% CI)
	Good (%)	Poor (%)		
Age				
20–29	84 (38.2)	136 (61.8)	0.81 (0.33–1.97)	2.22 (0.63–7.89)
30–39	16 (21.1)	60 (78.9)	1.875 (0.68–5.16)	4.78 (1.12–20.36)^a^
≥ 40	8 (33.3)	16 (66.7)	1.00	1.00
Educational status				
Grades 1–8	56 (43.8)	72 (56.2)	0.45 (0.28–0.72)	0.44 (0.25–0.76)
No schooling	52 (27.1)	140 (72.9)	1.00	1.00
Occupation (husband)				
Government employee	8 (33.3)	16 (66.7)	1.02 (0.42–2.47)	1.89 (0.66–5.42)
Self-employed	100 (33.8)	196 (66.2)	1.00	1.00
Household income				
Below 7895 ETB per month	80 (29.4)	192 (70.6)	3.36 (1.79–6.31)	6.41 (2.78–14.81)^a^
Above 7895 ETB per month	28 (58.3)	20 (41.7)	1.00	1.00
Pregnancy intention				
Wanted	84 (30.9)	188 (69.1)	2.24 (1.20–4.17)	2.63 (1.21–5.73)^a^
Unwanted	24 (50.0)	24 (50.0)	1.00	1.00
History of obstetric complication				
Yes	8 (9.1)	80 (90.9)	7.58 (3.50–16.39)	6.72 (2.81–16.07)^a^
No	100 (43.1)	132 (56.9)	1.00	1.00

^a^Statistically significant.

Furthermore, women in the age group (30–39 years) (AOR 4.23, 95% CI 1.14–15.65) were 4 times more likely to have a favorable attitude towards MWHs compared to women in the age group ≥ 40 years. Participants with household income below 7895 ETB (AOR 7.12, 95% CI 3.26–15.55) were 7 times more likely to have good attitude towards MWHs compared to those with household income above 7895 ETB. Similarly, women having wanted pregnancy (AOR 2.57, 95% CI 1.21–5.47) and those having a history of obstetric complications (AOR 5.59, 95% CI 2.30–13.59) were nearly 3 times and 6 times more likely to have good attitude towards MWHs compared to women having unwanted pregnancy and those had no history of obstetric complications, respectively (Table 5).

**Table 5:** Factors associated with women’s attitude towards MWHs in rural southern Ethiopia, 2022.

Variables	Level of attitude		Crude OR (95% CI)	Adjusted OR (95% CI)
	Favorable (%)	Unfavorable (%)		
Age				
20–29	68 (30.9)	152 (69.1)	1.12 (0.46, 2.74)	2.64 (0.81, 8.55)
30–39	16 (21.1)	60 (78.1)	1.88 (0.68, 5.16)	4.23 (1.14, 15.65)^a^
≥ 40	8 (33.3)	16 (66.7)	1.00	1.00
Educational status				
Grades 1–8	48 (35.3)	88 (64.7)	0.58 (0.35, 0.94)	1.66 (0.95, 2.91)
No formal schooling	44 (23.9)	140 (76.1)	1.00	1.00
Household income				
Below 7895 ETB per month	64 (23.5)	208 (76.5)	4.55 (2.41, 8.62)	7.12 (3.26, 15.55)^a^
Above 7895 ETB per month	28 (58.3)	20 (41.7)	1.00	1.00
Pregnancy intention				
Wanted	72 (26.5)	200 (73.5)	1.98 (1.05, 3.74)	2.57 (1.21, 5.47)^a^
Unwanted	20 (41.7)	28 (58.3)	1.00	1.00
History of obstetric complication				
Yes	8 (9.1)	80 (90.9)	5.68 (2.62, 12.32)	5.59 (2.30, 13.59)^a^
No	84 (36.2)	148 (63.8)	1.00	1.00

^a^Statistically significant.

## Discussion

This study was a cross-sectional analysis of baseline data from a cluster randomized trial conducted in rural Ethiopia. The participants were pregnant women in their second trimester. This analysis considered data collected on women’s experiences related to MWHs with the most recent pregnancy and birth before their current pregnancy. The findings showed that 33.75% had good knowledge of MWHs, 28.75% had favorable attitudes towards MWHs, 26.25% had good male partner involvement, and 23.75% used MWHs.

In this study, we found that around one-third (33.75%) of women had good knowledge about MWHs. Possible reasons that only one-third had good knowledge may be that most women who participated in this study had no formal education, low access to ANC, and poor access to health information due to long distances from health facilities. This finding is in line with studies from southern and central Ethiopia, which revealed that only 7% and 47.7% of women, respectively, had awareness about MWHs^28,29^. This finding contrasts another study from Ethiopia in which a significant proportion (87.7%) of the participants reported having awareness about MWHs^30^. We suggest one of the reasons for this difference may be the differences in health system related factors such as leadership, and access to health facilities including transportation; however, it requires further research. Furthermore, the current study showed that only 28.75% of the participants had favorable attitude towards MWHs. Possible reasons for this low-level attitude towards MWH may be poor ANC attendance, low institutional delivery practice, lack of awareness about MWH, and a low level of male partners’ participation in maternal health. Likewise, another study showed that less than half (48.8%) of women had favorable attitude towards using MWHs^31^, though this is still far from our finding. In contrast, a study showed that more than two-thirds (65.3%) of women had favorable attitude towards MWHs^32^. The possible reason for these differences may be sociodemographic or health system related factors.

The study revealed that the MWH utilization was 23.75%. The reasons for this low utilization of MWHs may be the low levels of good knowledge and favorable attitudes toward MWHs among the participants. Poor involvement of male partners in maternal health might also have affected women’s access to MWHs, as males are the main decision makers. In support of this, other studies from Ethiopia found that the MWH uptake was low^33,34^. The reason for this may be the observed similarity in participants educational status (i.e., majority of the participants had no formal education) in study settings; however, this requires further investigation. In contrast, another study from the Sidama Zone, Ethiopia, showed that more than two-thirds (67.25%) of women used MWHs^30^. One reason for this significant variance may be the differences in the level of awareness of MWHs. For example, in our study, only one-third of the women had good knowledge of MWHs, whereas in the above-mentioned study from the Sidama Zone^30^, more than three-fourths reported that they were aware of MWHs. Furthermore, a systematic review revealed that a lack of knowledge about MWHs was one of the reasons for the poor utilization of MWHs^35^. This finding implies that a lack of awareness may affect women’s access to MWHs. Therefore, improving maternal knowledge and attitude regarding MWH may improve access to MWHs among pregnant women in rural Ethiopia.

The current study showed that educational status was inversely associated with knowledge and not associated with attitudes towards MWHs. This might imply that those who have some formal schooling may have less concerns to the MWH services or were not using MWHs. This is consistent with findings from a study in Ethiopia^31^ whereas it contrasts with a study from Somaliland^36^. Further studies are needed to justify why having no or lower educational status was found to be associated with the knowledge of MWHs. Besides, in the current study, a history of obstetric complications and pregnancy intention (wanted pregnancy) were associated with knowledge of and attitudes toward MWHs. One reason may be that women who experienced pregnancy-related complications had painful experiences and, therefore, developed better health-seeking behavior and preferred to stay near health facilities to seek immediate obstetric care for their current pregnancy. Moreover, women who want to get pregnant may also have a better attitude towards staying at MWH, as they need newborns. Similarly, other studies have shown that a history of complications in previous childbirth and pregnancy intention was associated with the intention to use MWHs^28^. Educating women to have a planned pregnancy and providing them with information about MWH during health care visits may improve maternal knowledge and attitude towards MWHs. This may in turn improve maternal and newborn health outcomes by improving women’s access to MWHs.

### Limitations of the study

Recall bias may have affected our findings. Because the participants were asked about their experiences of last childbirth.

## Conclusion and recommendation

The majority of participants had poor knowledge of MWHs, unfavorable attitudes toward MWHs, and poor male partner involvement. The utilization of MWHs is low. Age, household income, pregnancy intention, and history of obstetric complications were associated with knowledge and attitude towards MWHs. Providing health education may improve women’s awareness and attitude towards MWHs. Age group and level of household income should be considered in planning and implementing MWH related behavioral interventions. Promoting male partner participation through educating couples may improve women’s access to MWHs.

## Supplementary Information

Supplementary Information.

## Data Availability

The data will be made available from the corresponding author up on request.
