# Supplementary material for: Factors associated with knowledge and attitude towards maternity waiting homes among pregnant women: baseline results from a cluster-randomized trial in rural Ethiopia
Source: Sci Rep. 2023 Jul 22;13:11854. doi: 10.1038/s41598-023-39029-1 (PMC10363115; doi:10.1038/s41598-023-39029-1)
Supplement: Supplementary file 1 — Supplementary Information. [file 41598_2023_39029_MOESM1_ESM.docx]

**Factors associated with knowledge and attitude towards maternity waiting homes among pregnant women: Baseline results from a cluster-randomized trial in rural Ethiopia**

Teklemariam Ergat Yarinbab, Hailay Abrha Gesesew, Margo Shawn Harrison, & Tefera Belachew

**Questionnaires: English Version**

Questionnaire ID Number ________

**Part I: Sociodemographic Characteristics**

| **S.No.** | **Questions** | **Response** | **Skip to …if** |
| --- | --- | --- | --- |
| Q101 | How old are you? | _________ years |  |
| Q102 | How old is your husband? | _________ years |  |
| Q103 | What is your ethnic group? | 1. Hadiya 2. Wolyta 3. Silte 4. Gurage 5. Others(specify) _ |  |
| Q104 | What is your husband’s ethnic group? | 1. Hadiya 2. Wolyta 3. Silte 4. Gurage 5. Others(specify) ___ |  |
| Q105 | What is your level of education? | 1. No formal education 2. Grade 1 – 8 3. Grade 9 – 12 4. College/University |  |
| Q106 | How many years of education have your husband completed? | 1. No formal education 2. Grade 1 – 8 3. Grade 9 – 12 4. College/University |  |
| Q107 | What is your husband’s occupation? | 1. Government employee 2. Farmer 3. NGO employee 4. Merchant 5. Student |  |
| Q108 | What is your occupation? | 1. Government employee 2. NGO employee 3. Merchant 4. Housewife 5. Student |  |
| Q109 | How much do you earn in a month? | _ ________ Birr/Month [Specify annual income if not feasible] |  |
| Q110 | How many children do you have? | ----------------------- |  |

**Part II: Obstetric Characteristics**

| S.No. | Questions | Response | Skip to --- if --- |
| --- | --- | --- | --- |
| Q201 | How many times have you been pregnant? | ------------------------- |  |
| Q202 | How many times have you gave birth to a fetus with gestational age of ≥24 weeks of gestation (both born alive & stillborn)? | ------------------------- |  |
| Q203 | What was the pregnancy intention with the last child? | 1. Wanted 2. Unwanted |  |
| Q204 | What was mode of delivery? | 1. Normal vaginal delivery 2. Caesarean section delivery 3. Other |  |
| Q205 | Did you experience any obstetric complication such as APH/PPH, Postpartum sepsis, Eclampsia, Ectopic pregnancy, Obstructed labor? Uterus rapture, etc. | 1. Yes 2. No |  |
| Q206 | What was the outcome of last pregnancy? | 1. Full term live birth 2. Preterm live birth 3. Still birth 4. Low birth weight 5. Other ------------- |  |
| Q207 | What was the gestational age at birth? | Gestational age ------------weeks |  |
| Q208 | What was the sex of newborn? | 1. Male 2. Female |  |

**Part III: Health service utilization**

| S.No. | Questions | Response | Skip to ---if--- |
| --- | --- | --- | --- |
| Q301 | How far is the closest health facility from your home? | 1. <2hrs of walk 2. ≥2hrs of walk |  |
| Q302 | Did you visit the health facility for ANC service in your last pregnancy? | 1. Yes 2. No |  |
| Q303 | Who make the decision for you to go to health facility for ANC? | 1. You 2. Your husband 3. Jointly [you & your husband] 4. Other (specify)----------------- |  |
| Q304 | Were you informed about your expected day of delivery during ANC visit? | 1. Yes 2. No |  |
| Q305 | Where did you give birth? | 1. At home 2. Health facility |  |
| Q306 | If you gave birth at health facility in your last pregnancy, then what form of transport did you use to go to the HF? | 1. Walking on foot 2. Carried by people 3. Ambulance 4. Animal back 5. Other (specify)--------- |  |
| Q307 | Did you pay for the transportation? | 1. Yes 2. No |  |
| Q308 | If yes to Q307, how much did you pay? | ------------- Birr |  |
| Q309 | How long did you wait to receive service at the facility? | 1. Receive the service immediately on arrival 2. Wait < 30 minutes 3. Wait > 30 minutes |  |
| Q310 | Were health workers respectful? | 1. Yes 2. No |  |
| Q311 | Was your husband with you at HF during labour and delivery? | 1. Yes 2. No |  |
| Q312 | Did you visit health facility for postnatal care? | 1. Yes 2. No |  |

**Part IV: MWH Utilization**

| S.No. | Questions | Responses | Skip to … if-- |
| --- | --- | --- | --- |
| Q401 | Have you ever heard about MWH? | 1. Yes 2. No |  |
| Q402 | If yes to Q#401, what was the source of information? | 1. Health Facility 2. HEW 3. Local radio 4. Other ------------------------- |  |
| Q403 | Is there MWH at your nearest HF? | 1. Yes 2. No 3. I don’t know |  |
| Q404 | Did you use MWH for your last pregnancy? | 1. Yes 2. No |  |
| Q405 | If yes for Q#404, who make the decision to use MWH? | 1. Yourself 2. Your husband 3. Jointly (you & your husband) 4. Health Professional 5. Relatives 6. Other (specify) ------------- |  |
| Q406 | What was the reason for your admission to MWH? | 1. Geographic distance 2. Previous CS repair 3. Previous obs fistula repair 4. Multiple pregnancy/ Multiparity 5. Previous stillbirth 6. Malpresentations 7. APH 8. Anemia 9. Other (specify) ----------------- |  |
| Q407 | Who was your attendant during your stay at MWH? | 1. Husband 2. Other family member/Relative 3. Other (specify) ---------------- |  |
| Q408 | Who provided financial support during your stay at MWH? [Transport, Food & Medications] | 1. Husband 2. Other family member/relative 3. Other(specify) ----------------- |  |
| Q409 | Who provided you social support during your stay at MWH? [Taking care of children, household care and/or other work, emotional support] | 1. Husband 2. Other family member/relative 3. Other (specify) ----------------------------------- |  |
| Q410 | How long did you stay at MWH? | -------------- days before delivery |  |

**Part V: Knowledge of MWHs**

| **S.No.** | **Questions** | **Yes** | **No** |
| --- | --- | --- | --- |
| Q501 | Knows what MWH is |  |  |
| Q502 | Knows importance of MWH |  |  |
| Q503 | Knows health facility with MWH |  |  |
| Q504 | Knows when to go to MWH |  |  |
| Q505 | Knows health services provided at MWH |  |  |
| Q506 | Knows women living in remote are recommended to stay at MWH |  |  |
| Q507 | Knows risky women are recommended to stay at MWH |  |  |

**Part VI: Attitude towards MWHs**

A five points Likert scale is used to measure maternal/paternal attitude. The numbers are labeled as follows: (1=strongly disagree) (2=disagree) (3=neutral) (4=agree) (5=strongly disagree)

| **S.No.** | **Attitude Statements** | **Scale** | | | | |
| --- | --- | --- | --- | --- | --- | --- |
|  |  | **1** | **2** | **3** | **4** | **5** |
| Q601 | MWH prevents distance barriers to obstetric care. | 1 | 2 | 3 | 4 | 5 |
| Q602 | MWH enables women to receive obstetric services timely during labor and delivery. | 1 | 2 | 3 | 4 | 5 |
| Q603 | Staying at MWH improves maternal health outcomes. | 1 | 2 | 3 | 4 | 5 |
| Q604 | I advise other pregnant women to stay at MWH. | 1 | 2 | 3 | 4 | 5 |
| Q605 | I will talk to my husband to allow me stay at MWH in future |  |  |  |  |  |

**Part VII: Male partners involvement**

| **S.No.** | **Activities** | **Yes** | **No** |
| --- | --- | --- | --- |
| Q701 | Did you talk with your husband about self-care during pregnancy in last pregnancy? |  |  |
| Q702 | Did you talk with your husband about place of birth in last childbirth? |  |  |
| Q703 | Did your husband take part in decision about ANC with you? |  |  |
| Q704 | Did your husband take part in decision about place of birth? |  |  |
| Q705 | Did your husband provide any type of financial support during pregnancy? |  |  |
| Q706 | Did your husband participated in doing household chores? |  |  |
| Q707 | Did your husband keep money aside for childbirth expenses? |  |  |
| Q708 | Did your husband accompany you to health facility for ANC? |  |  |
| Q709 | Did your husband accompany you to health facility for childbirth? |  |  |
